# Non-Suicidal Self-Injury and Emotion Regulation Strategies Among Secondary School and University Students: A Network Analysis Perspective

**DOI:** 10.3390/bs15111517

**Published:** 2025-11-08

**Authors:** Yang Yang, Tianyuan Ji, Yu Liu, Mingyangjia Tian, Yanan Yang, Yunyun Zhang, Lin Lin

**Affiliations:** 1 Collaborative Innovation Center of Assessment Toward Basic Education Quality, Beijing Normal University, Beijing 100875, China; 2 Faculty of Psychology, Tianjin Normal University, Tianjin 300387, China; 3 Key Research Base of Humanities and Social Sciences of the Ministry of Education, Academy of Psychology and Behavior, Tianjin Normal University, Tianjin 300387, China; 4 Department of Psychology, Zhejiang Normal University, Jinhua 321004, China; 5 Intelligent Laboratory of Child and Adolescent Mental Health and Crisis Intervention of Zhejiang Province, Zhejiang Normal University, Jinhua 321004, China; 6 Faculty of Psychology, Beijing Normal University, Beijing 100875, China; 7 Center of Cooperative Innovation for Assessment and Promotion of National Mental Health Under Ministry of Education, Tianjin Normal University, Tianjin 300387, China

**Keywords:** non-suicidal self-injury, emotion regulation strategies, network analysis, secondary school students, university students

## Abstract

Incidence of NSSI rises during adolescence and peaks in young adulthood. Secondary school and university students, representing these age groups, have been the focus of research on how emotion regulation strategies impact NSSI. However, a comprehensive study of the interrelations among different symptoms is needed. Research based on network analysis, a questionnaire survey on emotion regulation strategies and NSSI was conducted with 378 secondary school students and 593 first-year university students, all of whom reported a history of engagement in NSSI. The results indicated that Cognitive reappraisal symptoms showed a positive or no correlation with NSSI, while expressive suppression symptoms demonstrated a negative or no correlation. Secondary school and university students using cognitive reappraisal or expressive suppression also tended to use the other type of emotion regulation strategy simultaneously. In secondary school NSSI groups, core symptoms were linked to cognitive reappraisal, while in university groups, they were linked to expressive suppression. Intervention targets for NSSI in secondary school students included “I keep my emotions to myself”, and for university students, “I control my emotions by not expressing them.” Research reveals a complex mechanism underlying the link between NSSI and emotion regulation strategies in university and secondary school students, offering valuable insights for promoting the psychological health of adolescents and youths.

## 1. Introduction

Non-suicidal self-injury (NSSI) encompasses a variety of deliberate, self-inflicted behaviors that are not socially sanctioned and aim to damage body tissue without any suicidal intent (Nock & Favazza, 2009). Common forms of NSSI include cutting, biting, burning, preventing wound healing, and hitting or banging body parts (Swannell et al., 2014). Self-injury occurs at various stages of life development, being most common among young people. The incidence of NSSI starts to gradually increase from mid-adolescence to late adolescence and peaks during young adulthood (ages 18–34) (Gratz, 2001; Whitlock et al., 2011). Meta-analysis studies indicated that the prevalence of NSSI among middle school students in mainland China was 27.4%, while the incidence among university students was 16.6% (Pan et al., 2016; Han et al., 2017). NSSI is associated with a number of psychological disorders (Brausch & Gutierrez, 2010; Sinclair et al., 2010; Selby et al., 2012; Ammerman et al., 2016). Secondary school and university students, being key groups in adolescence and young adulthood, are in critical developmental transition phases. Therefore, it is essential to address the issue of NSSI in these populations and identify the factors influencing their NSSI behavior.

The use of emotion regulation strategies is considered to be closely related to NSSI. Emotion regulation strategies are habitual methods individuals use at various stages of emotional arousal to manage which emotions they experience and how they experience and express them (Gross, 1998a). The experiential avoidance model (Chapman et al., 2006) posits that self-injury is maintained through negative reinforcement. Individuals often engage in self-injury to avoid unpleasant emotional experiences and regulate their emotions. The lack of appropriate emotion regulation strategies is one of the reasons why individuals resort to avoidance behaviors, leading to self-injury. Research has found that alleviating negative emotions is the most commonly reported reason for NSSI, and some individuals with difficulties in emotion regulation report using NSSI to release emotional distress (Brown et al., 2002). When individuals repeatedly use the same method of NSSI, it indicates that this method has been effective in relieving their emotions, leading them to repeatedly attempt this NSSI method. Meanwhile, if an individual attempts a greater number of NSSI methods, this may indicate that previous methods failed to effectively regulate negative emotions. Consequently, there is a continuous need to seek new NSSI methods for emotional release. This persistent experimentation not only increases the frequency of NSSI but may also amplify feelings of helplessness and frustration, thereby elevating the risk of suicide (L. Lin et al., 2019). The number of NSSI methods an individual attempts not only reflects the frequency of past NSSI behaviors but also highlights the close relationship with the use of emotion regulation strategies. For individuals, adopting effective emotion regulation strategies can alleviate the negative emotions they experience, which helps to reduce NSSI behaviors stemming from emotional regulation difficulties. This, in turn, lowers the frequency of NSSI and decreases the number of NSSI methods used.

Moreover, different types of emotion regulation strategies are associated differently with NSSI. Expressive suppression and cognitive reappraisal are two of the most common emotion regulation strategies (Gross & John, 2003). Cognitive reappraisal involves changing one’s interpretation of emotional events, whereas expressive suppression involves inhibiting ongoing emotional expressive behaviors (Gross, 1998b, 2002). On one hand, cognitive reappraisal is negatively correlated with NSSI, as employing this strategy can more effectively alleviate individuals’ NSSI behaviors (Voon et al., 2014; Gu et al., 2022). On the other hand, unlike cognitive reappraisal, expressive suppression is positively correlated with NSSI, and individuals who engage in NSSI may be more inclined to use expressive suppression (Richmond et al., 2017; Hasking et al., 2010; Zhao et al., 2021). In summary, adopting effective emotion regulation strategies could help alleviate NSSI, and different strategies are likely to exert varying influences on NSSI.

Previous research on the relationship between NSSI and emotion regulation strategies often relied on overall scores to explain this relationship (Richmond et al., 2017; Xiong et al., 2023). This approach assumes that all symptoms (items) have equal importance, which may fail to fully account for the interactions between different symptoms of emotion regulation strategies and NSSI, potentially overlooking important details. Moreover, previous studies have mostly considered the association between the use of a single emotion regulation strategy and NSSI (Y. L. Wang et al., 2020; Gu et al., 2022). However, emotion regulation is complex and flexible; individuals often employ multiple strategies to regulate their emotions when faced with negative events, and there may be intricate interactions between different emotion regulation strategies (Brans et al., 2013; Mao et al., 2021). Hence, assessing a single emotion regulation strategy on its own is incomplete. The network analysis method can address these shortcomings. This analytical technique, developed based on dynamic systems models, presents information in the form of a network, primarily consisting of nodes representing observed variables and edges representing the statistical relationships between these variables. This method can identify core symptoms within the network (high-centrality symptoms) based on centrality indicators such as betweenness, closeness, and strength (Epskamp et al., 2018; Cai et al., 2020). The advantage of the network analysis method is that it can control for the relationships of other symptoms while evaluating the relationship between two symptoms (Blanken et al., 2019). Additionally, it can help identify bridge variables that directly link the development and maintenance of two problems, thereby enhancing the specificity of interventions (Garabiles et al., 2019). Therefore, this study chose to adopt the network analysis method to explore the relationship between emotion regulation strategies and NSSI.

### The Current Study

In previous research on the relationship between NSSI and emotion regulation strategies, a certain group of secondary school students or university students were mainly studied (Liu et al., 2022; Chen et al., 2023). However, different age groups exhibit distinct characteristics. Adolescents in secondary school are prone to impulsivity, and exhibit pronounced mood swings. During this period, studying is their primary task. In contrast, university students are in young adulthood, a stage often accompanied by complex emotional fluctuations and internal challenges, with emotions frequently being concealed. At this time, they may be exploring their future direction (C. D. Lin, 2009). These characteristics may influence their use of emotion regulation strategies. Therefore, conducting group-specific research on their respective behavioral patterns and related mechanisms is crucial for the targeted prevention of NSSI.

This study simultaneously focused on both secondary school and university student groups. Using the network analysis method, we established symptom networks for NSSI and emotion regulation strategies among secondary school and university students. This approach aimed to explore the network structures of NSSI and emotion regulation strategies in these two groups. By comparing the characteristics of NSSI and emotion regulation strategies between these groups, we sought to identify the connections between NSSI and emotion regulation strategies. This would provide targeted intervention strategies and theoretical foundations for understanding the mechanisms of NSSI in secondary school and university students. Additionally, based on the association between the number of NSSI methods and emotion regulation, this study also included the number of NSSI methods as one of the symptoms in the network.

## 2. Methods

### 2.1. Participants and Procedures

The study participants were recruited from a secondary school and a university in Tianjin, China. We contacted the secondary school and the university offline, and conducted a questionnaire survey among first-year university students, 10th-grade students, and 7th-grade students from both schools. The survey was conducted in September 2019. We also excluded invalid questionnaires (e.g., those with incorrect responses to validity check items or with excessive missing values). A total of 798 questionnaires were distributed to secondary school students, with 765 valid responses (421 from grade 7 and 344 from grade 10), yielding an effective rate of 95.86%. Among them, 378 students reported having engaged in NSSI at least once (213 from grade 7 and 165 from grade 10), including 176 males, 199 females, and 3 individuals who did not specify their gender. The average age was 13.66 years (*SD* = 1.54). A total of 2292 questionnaires were distributed to university students, with 2261 valid responses, resulting in an effective rate of 98.65%. Among them, 593 students reported having engaged in NSSI at least once, including 145 males and 448 females. The average age was 18.27 years (*SD* = 0.70). The first language of all participants was Chinese. Detailed demographic information is presented in Table 1.

First, we distributed questionnaires to secondary school students by class. Second, we conducted a questionnaire survey among first-year university students who were enrolled in public elective courses using cluster sampling. Both surveys were administered by graduate students majoring in psychology who had received professional training. Prior to the commencement of the test, the main experimenter briefed the participants on the response requirements and emphasized the confidentiality of their personal information. Each participant read and signed an informed consent form. For participants under the age of 18, we obtained the informed consent of the individuals themselves and their guardians. After completing the survey, all participants were provided with contact information for local mental health hotlines and received a small gift as a token of appreciation. This study selected participants with a history of NSSI for analysis. The study was approved by Ethics Committee of the Academy of Psychology and Behavior of Tianjin Normal University.

### 2.2. Measures

#### 2.2.1. Non-Suicidal Self-Injury

The Adolescents Self-Harm Scale (ASHS), developed by Zheng (2006) and revised by Feng (2008), was used to assess NSSI among secondary school and university students over the past six months. This questionnaire consists of 18 items, which evaluate both the frequency and the severity of self-injury. The frequency of self-injury is rated on a four-level scale, while the severity is rated on a five-level scale. The total score is calculated as the sum of the products of the frequency and severity scores for each item. A higher total score indicates more severe NSSI behaviors, whereas a total score of zero indicates the absence of NSSI behaviors. In order to obtain the number of NSSI methods used by the participants, we converted the scoring of each NSSI method corresponding to its frequency of self-injury into a binary score (0 points if the NSSI method was not attempted, and 1 point if the NSSI method was attempted). Then, the scores for each participant were summed to obtain the score for the number of attempted NSSI methods. The Cronbach’s alpha coefficient of this questionnaire was 0.85. In the current study, the Cronbach’s alpha coefficient of the secondary school students was 0.86, and for the university students group, it was 0.87.

#### 2.2.2. Emotion Regulation Strategies

Cognitive reappraisal and expressive suppression were assessed using the Emotion Regulation Questionnaire (Gross, 1998a). The Chinese version was revised by L. Wang et al. (2007). This questionnaire consists of 10 items with two subscales: cognitive reappraisal with 6 items and expressive suppression with 4 items, all rated on a 7-point scale. Higher scores on the subscales indicate a higher frequency of using the respective emotion regulation strategy. In this questionnaire, the Cronbach’s alpha coefficient for the cognitive reappraisal subscale was 0.85, and for the expressive suppression subscale, it was 0.77. In the present study, the Cronbach’s alpha coefficient for the cognitive reappraisal subscale was 0.83 and for the expressive suppression subscale was 0.70 among secondary school students; while among university students, the Cronbach’s alpha coefficient for the cognitive reappraisal subscale was 0.87 and for the expressive suppression subscale was 0.74.

### 2.3. Data Analysis

First, we performed descriptive statistics in SPSS 26.0. Second, using the qgraph package in R 4.2.1, we constructed a network model involving the severity of NSSI, the frequency of NSSI, and the number of NSSI methods with the 10 items of emotion regulation strategies (Epskamp et al., 2012). We estimated an EBIC graphical LASSO network model with 13 items, which allowed every association between two symptoms to be controlled for all the symptoms after applying a LASSO regularization (Epskamp & Fried, 2018; Van den Bergh et al., 2021). Subsequently, we calculated centrality indices to compare the importance of each node within the network. Research has shown that strength is more stable and reliable than betweenness and closeness, making it more suitable for assessing node centrality in psychological networks (Bringmann et al., 2019). Therefore, we chose strength, which is the sum of the absolute weights of the edge connecting the node to all the other nodes, to compare the importance of nodes within the network. A higher strength value indicates greater centrality of the node within the entire network (Borsboom & Cramer, 2013). Thirdly, we utilized the bootnet package to calculate the correlation stability (CS) coefficient through the bootstrap sampling method (using 1000 bootstrap samples). The CS coefficient is used to assess the stability of node strength, where a CS coefficient ≥ 0.50 indicates a certain level of stability (Epskamp et al., 2018). Lastly, in addition to computing the strength centrality of the network, we employed the networktools package to calculate the expected influence of each node as bridge expected influence, thereby identifying bridge nodes.

## 3. Results

### 3.1. The Network Models of NSSI and Emotion Regulation Strategies

**Figure 1:**
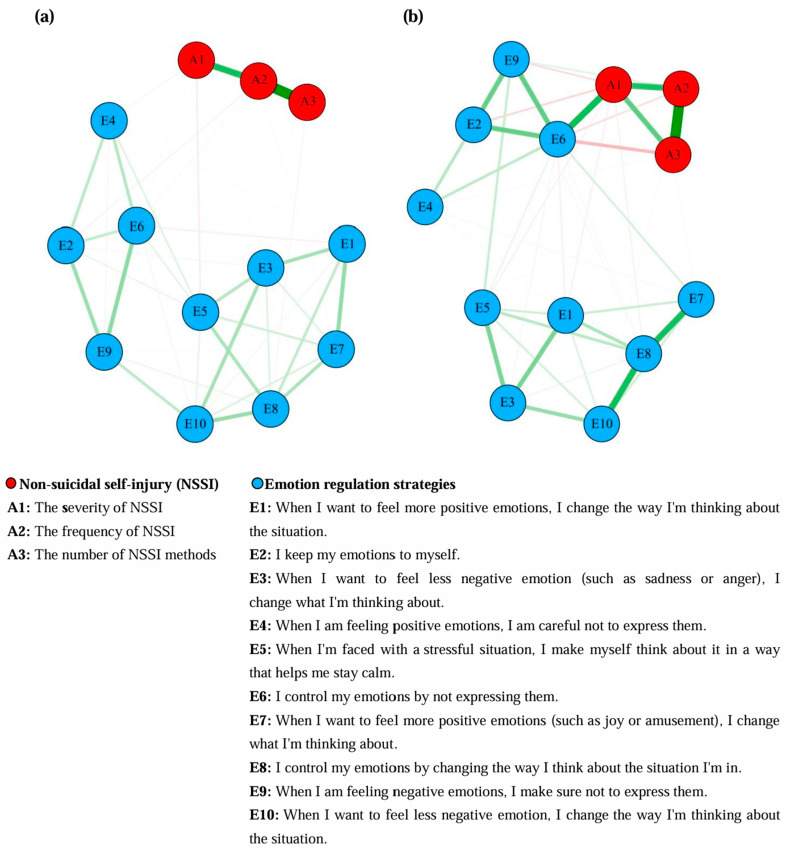
Network structure models of NSSI and emotion regulation strategies for secondary school and university students. Green lines represent positive correlations, and red lines represent negative correlations. The thickness of the lines indicates the strength of the correlations. (**a**) The network structure based on the group of 378 secondary school students. (**b**) The network structure based on the group of 593 university students.

Figure 1 shows the network model of NSSI and emotion regulation strategies for secondary school students and university students, respectively. To begin with, based on the analysis results of the secondary school student group (Figure 1a), the within-group analysis showed that symptoms A2 and A3 had the strongest connection strength (0.78). Symptoms E6 and E9 had the strongest positive correlation (0.28), followed by symptoms E1 and E7 (0.27). Symptoms E1 and E6 had the strongest negative correlation (−0.05). Additionally, the results also showed that symptoms E4 and E5 had a relatively strong positive correlation (0.09). In the between-group analysis, symptom A1 was positively correlated with symptoms E4 and E6 (0.03 and 0.01, respectively), and negatively correlated with symptoms E1 and E5 (−0.01 and −0.06, respectively). Symptom A2 was positively correlated with symptoms E2, E6, and E7 (0.04, 0.03, and 0.02, respectively). Symptom A3 was negatively correlated with symptoms E5 and E8 (−0.01 and −0.03, respectively).

Next, based on the analysis results of the university student group (Figure 1b), the within-group analysis showed that symptoms A2 and A3 also had the strongest connection strength (0.66). Symptoms E8 and E10 had the strongest positive correlation (0.41), followed by symptoms E7 and E8 (0.40). Moreover, symptoms E5 and E9 also had a relatively strong positive correlation (0.12). Symptoms E1 and E6 had the strongest negative correlation (−0.05). In the between-group analysis, symptom A1 was positively correlated with symptom E6 (0.42), and negatively correlated with symptoms E1, E2, E5, E8, and E9 (−0.04, −0.10, −0.04, −0.04, and −0.09, respectively). Symptom A2 was positively correlated with symptom E9 (0.07), and negatively correlated with symptoms E6 and E10 (−0.08 and −0.02, respectively). Symptom A3 was positively correlated with symptoms E2, E4, and E7 (0.05, 0.02, and 0.02, respectively), and negatively correlated with symptoms E5 and E6 (−0.01 and −0.18, respectively).

### 3.2. The Central Nodes in the Networks of NSSI and Emotion Regulation Strategies

**Figure 2:**
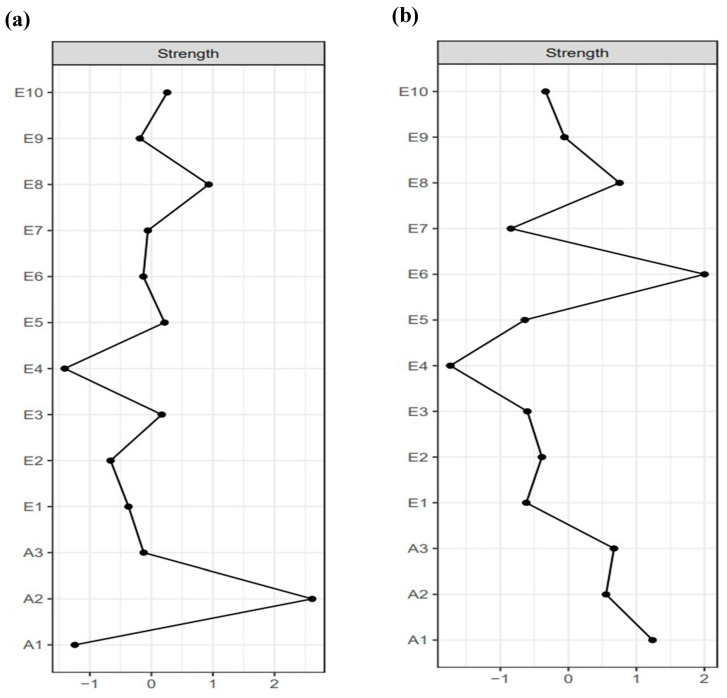
Centrality indicators (Strength) for NSSI and Emotion Regulation Strategies in secondary school and university students. (**a**) Strength value for the secondary school student network; (**b**) Strength value for the university student network. Full names of the abbreviations can be found in Figure 1.

Subsequently, this study calculated the centrality of the network nodes. The results are shown in Figure 2. Figure 2a shows the strength centrality of the network of NSSI and emotion regulation strategies for secondary school students. It was found that symptom A2, “The frequency of NSSI,” had the highest strength, followed by symptom E8 “I control my emotions by changing the way I think about the situation I’m in.” The CS coefficient for node strength was 0.67, which is greater than 0.50. Figure 2b shows the strength centrality of the network of NSSI and emotion regulation strategies for university students. The results showed that symptom E6, “I control my emotions by not expressing them,” had the highest strength, followed by symptom A1, “The severity of NSSI.” The CS coefficient for node strength was 0.75, also greater than 0.50. These results indicated that the central nodes in the networks of both secondary school and university students exhibited good stability.

### 3.3. The Bridging Nodes in the Networks of NSSI and Emotion Regulation Strategies

**Figure 3:**
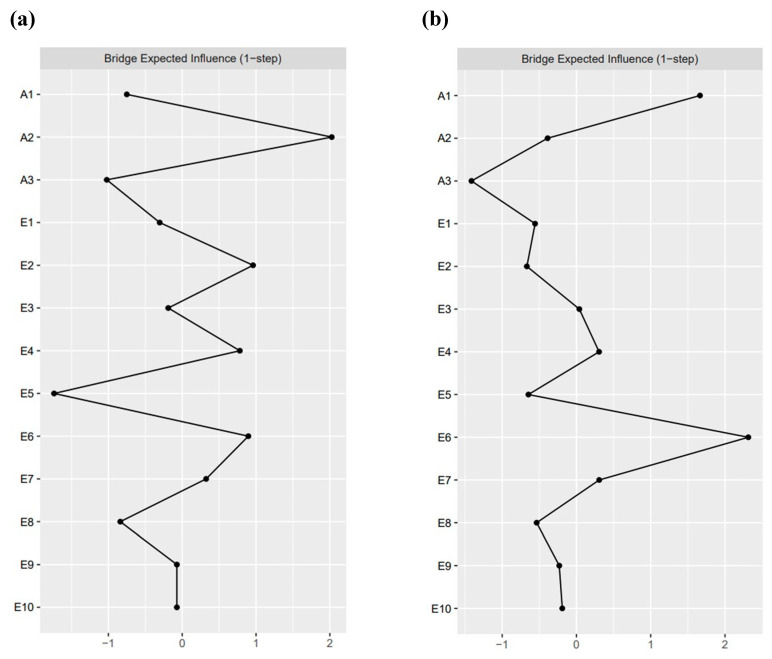
Bridge centrality indicators for NSSI and emotion regulation strategies in secondary school and university students. (**a**) Bridge expected influence in the secondary school student network; (**b**) bridge expected influence in the university student network. Full names of the abbreviations can be found in Figure 1.

Figure 3 shows the bridge centrality indicators for the networks of NSSI and emotion regulation strategies for secondary school and university students. In the secondary school NSSI group (Figure 3a), symptom A2, “The frequency of NSSI,” had the highest bridge expected influence, indicating the strongest association with emotion regulation strategies. Within the secondary school emotion regulation strategy group, symptom E2, “I keep my emotions to myself,” had the highest bridge expected influence and showed the strongest association with NSSI. In the university NSSI group (Figure 3b), symptom A1, “The severity of NSSI,” had the highest association with emotion regulation strategies; within the university emotion regulation strategy group, symptom E6, “I control my emotions by not expressing them,” had the highest association with NSSI.

## 4. Discussion

The risk of NSSI cannot be overlooked, as it may lead to various adaptive problems. Therefore, identifying protective factors against NSSI is crucial. Emotion regulation strategies, as influential factors of NSSI, have garnered significant attention. Previous studies have explored the relationship between NSSI and emotion regulation strategies (Hasking et al., 2010; Gu et al., 2022). Building on these studies, this research employed network analysis to examine the connections between NSSI and different components of emotion regulation strategies among secondary school students and university students. The study further investigated the mechanisms of NSSI in different groups and aimed to identify targeted intervention measures.

Firstly, our research results indicated that both “the frequency of NSSI” and “the number of NSSI methods” had a strong positive correlation in both the secondary school student group and the university student group. This suggests that the more methods of NSSI an individual employs, the more NSSI experiences they have, that is, the higher the frequency of NSSI. This finding is consistent with previous research (L. Lin et al., 2019), which posited that the number of NSSI methods influences the frequency of NSSI. Subsequently, our research results also indicated that when the symptoms of emotion regulation strategies are detailed, the impact of various symptoms under emotion regulation strategies on NSSI behavior is not the same for both secondary school students and university students. Although previous research has shown that cognitive reappraisal strategies are beneficial for alleviating NSSI, while expressive suppression strategies may exacerbate NSSI behavior (Johnson et al., 2016; Richmond et al., 2017), these studies mostly used overall scores for their examinations. The current study found that not all symptoms within cognitive reappraisal strategies are negatively associated with NSSI. Some symptoms showed no association, while others exhibited a positive correlation. For instance, in the secondary school student group, A2, “The frequency of NSSI,” was positively correlated with E7, “When I want to feel more positive emotions (such as joy or amusement), I change what I’m thinking about,” and in the university student group, A3, “The number of NSSI methods,” was positively correlated with E7, “When I want to feel more positive emotions (such as joy or amusement), I change what I’m thinking about.” Similarly, not all symptoms within expressive suppression were positively associated with NSSI. Some symptoms exhibited a negative correlation with NSSI, such as in the university student group network model where A3, “The number of NSSI methods,” was negatively correlated with E6, “I control my emotions by not expressing them,” while some symptoms showed no association with NSSI.

Additionally, this study found that cognitive reappraisal and expressive suppression, as factors influencing NSSI, did not exhibit a completely inverse relationship. Secondary school students and university students who frequently use cognitive reappraisal or expressive suppression may also be inclined to simultaneously employ the other type of strategy (such as expressive suppression or cognitive reappraisal). For example, in the network model of secondary school students, E4, “When I’m feeling positive emotions, I’m careful not to express them,” was positively associated with E5, “When I’m faced with a stressful situation, I make myself think about it in a way that helps me stay calm.” In the network model of university students, E5 was also positively associated with E9, “When I’m feeling negative emotions, I make sure not to express them.” Aldao and Nolen-Hoeksema (2013) pointed out that emotion regulation is a complex process. When faced with emotionally evocative stimuli, individuals are not limited to adopting a single emotion regulation strategy; instead, they may employ multiple strategies. When an individual finds that one emotion regulation strategy does not achieve the desired result, they often try other strategies. Both positive strategies (such as cognitive reappraisal) and negative strategies (such as expressive suppression) may be chosen. Research indicated that individuals engaged in NSSI not only in situations where they were dealing with negative emotions, but also in positive emotional responses (Perini et al., 2021), suggesting that the type of emotion plays a complex role in triggering NSSI (Boyes et al., 2020). Therefore, when exploring the relationship between NSSI and emotion regulation strategies, it is important to take into account the complexity of emotional influences on NSSI and the interactions among different emotion regulation strategies.

Secondly, the results showed that E8, “I control my emotions by changing the way I think about the situation I’m in,” occupied a significant central position in the network model of the NSSI group among secondary school students, while E6, “I control my emotions by not expressing them,” held a crucial central position in the network model of the NSSI group among university students. In the secondary school student group, the core symptom belonged to the cognitive reappraisal strategy, while in the university student group, the core symptom was the expressive suppression strategy. Compared to secondary school students, university students face not only academic pressure and interpersonal relationship issues but also the challenges of entering society. Consequently, they often endure greater stress. At this stage, students have passed through adolescence and entered young adulthood, undergoing physical and mental changes that result in stronger self-control and more restrained emotions (Chu, 2005). Therefore, they may be more likely to use expressive suppression strategies. In the secondary school student group, the core symptom belonged to the cognitive reappraisal strategy, while in the university student group, the core symptom was the expressive suppression strategy. The activation of core symptoms in secondary school students and university students might lead to a more widespread activation of the entire network. Therefore, this result suggests that for secondary school students, particularly those with NSSI behaviors, it is important to promote the development of cognitive reappraisal. For university students, attention should be paid to their use of expressive suppression.

Lastly, this study also identified the bridge nodes within the network models of secondary school and university students. In the network structure of secondary school students, E2, “I keep my emotions to myself,” exhibited the strongest bridging relationship with NSSI. In the network structure of university students, E6, “I control my emotions by not expressing them,” had the strongest bridging relationship with NSSI. On the one hand, in general, both bridge nodes fall under the category of expressive suppression strategies, indicating that expressive suppression is a risk factor for NSSI. This finding is consistent with previous research (Tan et al., 2023; Zeng et al., 2024). According to the experiential avoidance model (Chapman et al., 2006), the avoidance mentality arising from the inability to eliminate negative emotions is a core factor leading to NSSI. The use of expressive suppression strategies does not completely eliminate negative emotions (Cheng et al., 2009). Therefore, the use of expressive suppression strategies is more likely to induce avoidance mentality, thereby leading to NSSI. On the other hand, when intervening in NSSI among secondary school and university students, it is important to target “I keep my emotions to myself” and “I control my emotions by not expressing them”, respectively, based on their developmental characteristics. Compared to other symptoms, these targets can have a more comprehensive and effective impact on NSSI. The current research findings suggest that future studies should not only examine the overall effects but also consider the interrelationships between various symptoms. This approach will provide a more accurate reflection of the relationship between emotional regulation strategies and NSSI, thereby identifying the influencing factors of NSSI more precisely.

## 5. Limitations and Future Directions

Firstly, the measurements in this study relied on self-reports from participants, which may be subject to social desirability bias. Future research could utilize multi-informant assessment methods to obtain more objective data. Secondly, this study was cross-sectional, making it impossible to explore the changes in NSSI and emotional regulation strategies over time among secondary school and university students. Future studies should conduct longitudinal tracking to analyze the longitudinal network of NSSI and emotional regulation strategies in these groups. Thirdly, although this study separately examined secondary school and university student groups, the sample sizes for both groups were relatively small. Future research should expand the sample size to conduct more detailed studies.

## 6. Conclusions

This study examined the relationships between various symptoms of NSSI and emotion regulation strategies, revealing the complex interactions between NSSI and emotion regulation strategies in both university and secondary school students. First, the influence of emotion regulation strategies on NSSI was complex, with certain symptoms of cognitive reappraisal positively or not correlated with NSSI, while certain symptoms of expressive suppression were negatively or not correlated with NSSI. Additionally, students who frequently used cognitive reappraisal or expressive suppression in NSSI might also have tended to simultaneously use the other type of emotion regulation strategy (expressive suppression or cognitive reappraisal). Second, within the network of NSSI and emotion regulation strategies, cognitive reappraisal emerged as the core symptom for NSSI among secondary school students, while expressive suppression was the core symptom among university students. Lastly, “I keep my emotions to myself” in secondary school students and “I control my emotions by not expressing them” in university students were the bridging nodes of their NSSI behaviors and important targets for intervention. Future research should further explore the interrelationships between different factors influencing NSSI to more precisely identify the key determinants of these behaviors.

## Figures and Tables

**Table 1:** Demographic Information of Participants.

	Participant Information	Secondary School Student (*n* = 378)		High School Student (*n* = 593)	
		*n*	%	*n*	%
Sex	Female	176	46.56	145	24.45
	Male	199	52.65	448	75.55
	Missing	3	0.79	0	0
Location	City	246	65.07	447	75.37
	Town	99	26.19	78	13.15
	Village	27	7.14	68	11.47
	Missing	6	1.59	0	0
Family structure	Nuclear family	257	67.99	421	70.99
	Extended family	67	17.72	112	18.89
	Not living with parents	17	4.50	11	1.85
	Single-parent family	31	8.20	49	8.26
	Missing	6	1.59	0	0
Economic status	High	60	15.87	67	11.13
	Average	268	70.90	100	67.45
	Low	38	10.06	127	21.42
	Missing	12	3.17	0	0

## Data Availability

The data presented in this study are available on request from the corresponding author. The data are not publicly available due to ethical and privacy concerns.

## References

[B1-behavsci-15-01517] Aldao A., Nolen-Hoeksema S. (2013). One versus many: Capturing the use of multiple emotion regulation strategies in response to an emotion-eliciting stimulus. Cognition & Emotion.

[B2-behavsci-15-01517] Ammerman B. A., Jacobucci R., Kleiman E. M., Muehlenkamp J. J., Mccloskey M. S. (2016). Development and validation of empirically derived frequency criteria for NSSI disorder using exploratory data mining. Psychological Assessment.

[B3-behavsci-15-01517] Blanken T. F., Van Der Zweerde T., Van Straten A., Van Someren E. J. W., Borsboom D., Lancee J. (2019). Introducing network intervention analysis to investigate sequential, symptom-specific treatment effects: A demonstration in co-occurring insomnia and depression. Psychotherapy and Psychosomatics.

[B4-behavsci-15-01517] Borsboom D., Cramer A. O. J. (2013). Network analysis: An integrative approach to the structure of psychopathology. Annual Review of Clinical Psychology.

[B5-behavsci-15-01517] Boyes M. E., Wilmot A., Hasking P. A. (2020). Nonsuicidal self-injury-related differences in the experience of negative and positive emotion. Suicide & Life-Threatening Behavior.

[B6-behavsci-15-01517] Brans K., Koval P., Verduyn P., Lim Y. L., Kuppens P. (2013). The regulation of negative and positive affect in daily life. Emotion.

[B7-behavsci-15-01517] Brausch A. M., Gutierrez P. M. (2010). Differences in non-suicidal self-injury and suicide attempts in adolescents. Journal of Youth and Adolescence.

[B8-behavsci-15-01517] Bringmann L. F., Elmer T., Epskamp S., Krause R. W., Schoch D., Wichers M., Wigman J. T. W., Snippe E. (2019). What do centrality measures measure in psychological networks?. Journal of Abnormal Psychology.

[B9-behavsci-15-01517] Brown M. Z., Comtois K. A., Linehan M. M. (2002). Reasons for suicide attempts and nonsuicidal self-injury in women with borderline personality disorder. Journal of Abnormal Psychology.

[B10-behavsci-15-01517] Cai Y. Q., Dong S. Y., Yuan S., Hu C. P. (2020). Network analysis and its applications in psychology. Advances in Psychological Science.

[B11-behavsci-15-01517] Chapman A. L., Gratz K. L., Brown M. Z. (2006). Solving the puzzle of deliberate self-harm: The experiential avoidance model. Behaviour Research & Therapy.

[B12-behavsci-15-01517] Chen Z. X., Li J. W., Wang Y. M., Liu X. (2023). The relationship between cumulative environmental risk and non-suicidal self-injury among college students: The effects of emotion regulation strategies. Psychological Development and Education.

[B13-behavsci-15-01517] Cheng L., Yuan J. J., He Y. Y., Li H. (2009). Emotion regulation strategies: Cognitive reappraisal is more effective than expressive suppression. Advances in Psychological Science.

[B14-behavsci-15-01517] Chu W. (2005). Analysis about the feature and training of self- consciousness in modern college students. Medicine and Philosophy.

[B16-behavsci-15-01517] Epskamp S., Borsboom D., Fried E. I. (2018). Estimating psychological networks and their accuracy: A tutorial paper. Behavior Research Methods.

[B17-behavsci-15-01517] Epskamp S., Cramer A. O. J., Waldorp L. J., Schmittmann V. D., Borsboom D. (2012). Qgraph: Network visualizations of relationships in psychometric data. Journal of Statistical Software.

[B15-behavsci-15-01517] Epskamp S., Fried E. I. (2018). A tutorial on regularized partial correlation networks. Psychological Methods.

[B18-behavsci-15-01517] Feng Y. (2008). The relation of adolescents’ self-harm behaviors, individual emotion characteristics and family environment factors.

[B19-behavsci-15-01517] Garabiles M. R., Lao C. K., Xiong Y., Hall B. J. (2019). Exploring comorbidity between anxiety and depression among migrant Filipino domestic workers: A network approach. Journal of Affective Disorders.

[B20-behavsci-15-01517] Gratz K. L. (2001). Measurement of deliberate self-harm: Preliminary data on the deliberate self-harm inventory. Journal of Psychopathology & Behavioral Assessment.

[B21-behavsci-15-01517] Gross J. J. (1998a). The emerging field of emotion regulation: An integrative review. Review of General Psychology.

[B22-behavsci-15-01517] Gross J. J. (1998b). Antecedent- and response-focused emotion regulation: Divergent consequences for experience, expression, and physiology. Journal of Personality and Social Psychology.

[B23-behavsci-15-01517] Gross J. J. (2002). Emotion regulation: Affective, cognitive, and social consequences. Psychophysiology.

[B24-behavsci-15-01517] Gross J. J., John O. P. (2003). Individual differences in two emotion regulation processes: Implications for affect, relationships, and well-being. Journal of Personality and Social Psychology.

[B25-behavsci-15-01517] Gu H. L., Ding Z. Y., Xia T. S., Wang L. Z. (2022). Self-criticism and adolescent non-suicidal self-injury: Moderated mediation effect. Psychological Development and Education.

[B26-behavsci-15-01517] Han A. Z., Xu G., Su P. Y. (2017). A meta-analysis of characteristics of non-suicidal self-injury among middle school students in mainland China. Chinese Journal of School Health.

[B27-behavsci-15-01517] Hasking P. A., Coric S. J., Swannell S., Martin G., Thompson H. K., Frost A. D. J. (2010). Brief report: Emotion regulation and coping as moderators in the relationship between personality and self-injury. Journal of Adolescence.

[B28-behavsci-15-01517] Johnson J., Oconnor D. B., Jones C. E., Jackson C., Hughes G., Ferguson E. (2016). Reappraisal buffers the association between stress and negative mood measured over 14 days: Implications for understanding psychological resilience. European Journal of Personality.

[B29-behavsci-15-01517] Lin C. D. (2009). Developmental psychology.

[B30-behavsci-15-01517] Lin L., He H. P., Liu Y., Yang Y., Mo J. C., Wang C. X., Liu T. (2019). Many times and how many ways: The impact of number of the impacts of negative emotion on suicide risk: The mediating effect of self –injury. Heilongjiang Researches on Higher Education.

[B31-behavsci-15-01517] Liu Z. J., Zhou F., Zheng Z. W., Li J. Y., Xiong Y. N., Huang P. (2022). Relationships between being bullied and non-suicidal self-injury among 2040 middle school students: The mediating role of emotion regulation strategies. Injury Medicine (Electronic Edition).

[B32-behavsci-15-01517] Mao C., Li Y. Y., Zhang Y. X., Xie Z. H., Li P. (2021). The relationship between alexithymia and cognitive emotion regulation strategies in nursing college students: A network analysis model. Chinese Journal of Clinical Psychology.

[B33-behavsci-15-01517] Nock M. K., Favazza A. R., Nock M. K. (2009). Nonsuicidal self-injury: Definition and classification. Understanding non-suicidal self-injury: Origins, assessment and treatment.

[B34-behavsci-15-01517] Pan Z., Mao S. J., Tang H. M., Fu Y. Y., Sun W. X., Liao Z. L., Li J. N., Qiu H. H., Zhu J. Y., Huang P. (2016). Meta-analysis on non-suicidal self-injury among college students in China. Chinese Journal of School Health.

[B35-behavsci-15-01517] Perini I., Zetterqvist M., Mayo L. M. (2021). Beyond distress: A role for positive affect in nonsuicidal self-injury. Current Opinion in Behavioral Sciences.

[B36-behavsci-15-01517] Richmond S., Hasking P., Meaney R. (2017). Psychological distress and non-suicidal self-injury: The mediating roles of rumination, cognitive reappraisal, and expressive suppression. Archives of Suicide Research.

[B37-behavsci-15-01517] Selby E. A., Bender T. W., Gordon K. H., Nock M. K., Joiner T. E. (2012). Non-suicidal self-injury (NSSI) disorder: A preliminary study. Personality Disorders.

[B38-behavsci-15-01517] Sinclair J. M. A., Hawton K., Gray A. (2010). Six year follow-up of a clinical sample of self-harm patients. Journal of Affective Disorders.

[B39-behavsci-15-01517] Swannell S. V., Martin G. E., Page A., Hasking P., St John N. J. (2014). Prevalence of nonsuicidal self-injury in nonclinical samples: Systematic review, meta-analysis and meta-regression. Suicide and Life-Threatening Behavior.

[B40-behavsci-15-01517] Tan H. X., Lin S. Y., Long Y. C., Zeng C. (2023). Relationship between childhood emotional abuse and self-harm behavior in adolescents: A moderated mediation analysis. Psychological Exploration.

[B41-behavsci-15-01517] Van den Bergh N., Marchetti I., Koster E. H. W. (2021). Bridges over troubled waters: Mapping the interplay between anxiety, depression and stress through network analysis of the DASS-21. Cognitive Therapy and Research.

[B42-behavsci-15-01517] Voon D., Hasking P., Martin G. (2014). Change in emotion regulation strategy use and its impact on adolescent nonsuicidal self-injury: A three year longitudinal analysis using latent growth modeling. Journal of Abnormal Psychology.

[B43-behavsci-15-01517] Wang L., Liu H. C., Li Z. Q., Du W. (2007). Reliability and validity study of the Chinese version of emotion regulation questionnaire. Chinese Journal of Health Psychology.

[B44-behavsci-15-01517] Wang Y. L., Chen H. L., Yuan Y. (2020). The impact of social exclusion on adolescent self-harm: The mediating role of shame and the moderating role of cognitive reappraisal. Psychological Science.

[B45-behavsci-15-01517] Whitlock J., Muehlenkamp J., Purington A., Eckenrode J., Barreira P., Baral Abrams G., Marchell T., Kress V., Girard K., Chin C., Knox K. (2011). Nonsuicidal self-injury in a college population: General trends and sex differences. Journal of American College Health.

[B46-behavsci-15-01517] Xiong Q., Shen X. Y., Yang H., Wang X. H., Yi J. Y., Zhu X. Z. (2023). Alexithymia and adolescents’ non-suicidal self-injury: The chain mediating of maladaptive emotion regulation and depression. Chinese Journal of Clinical Psychology.

[B47-behavsci-15-01517] Zeng C., Yu C., Pu W. D., Liao S. Q. (2024). The impact of parent-child alienation on adolescents’non-suicidal self-injury: A moderated mediating model. Chinese Journal of Clinical Psychology.

[B48-behavsci-15-01517] Zhao T. X., Zhong Y. J., Wei Y. J., Su Y. L., Dang Y. H., Wu X. L. (2021). Emotion regulation strategies and family function in non-suicidal self-injury adolescents. Chinese Journal of Child Health Care.

[B49-behavsci-15-01517] Zheng Y. (2006). Epidemiologic investigation of self-mutilation behavior among adolescents in Wuhan and its functional model.

